# Multiplexed and amplified chemiluminescence resonance energy transfer (CRET) detection of genes and microRNAs using dye-loaded hemin/G-quadruplex-modified UiO-66 metal–organic framework nanoparticles†

**DOI:** 10.1039/d0sc06744j

**Published:** 2021-02-08

**Authors:** Pu Zhang, Yu Ouyang, Itamar Willner

**Affiliations:** Institute of Chemistry, Center for Nanoscience and Nanotechnology, The Hebrew University of Jerusalem Jerusalem 91904 Israel itamar.willner@mail.huji.ac.il

## Abstract

Dye-loaded UiO-66 metal–organic framework nanoparticles (NMOFs) modified with catalytic hemin/G-quadruplex DNAzyme labels act as functional hybrid modules for the chemiluminescence resonance energy transfer (CRET) analysis of miRNAs (miRNA-155 or miRNA-21) or genes (p53 or BRCA1). The dye-loaded NMOFs (dye = fluorescein (Fl) or rhodamine 6G (Rh 6G)) are modified with hairpin probes that are engineered to include in their loop domains recognition sequences for the miRNAs or genes, and in their stem regions caged G-quadruplex domains. In the presence of the analytes miRNAs or genes, the hairpin structures are opened, leading, in the presence of hemin, to the self-assembly of hemin/G-quadruplex DNAzyme labels linked to the dye-loaded NMOFs. In the presence of luminol and H_2_O_2_, the hemin/G-quadruplex DNAzyme labels catalyze the generation of chemiluminescence that provides radiative energy to stimulate the process of CRET to the dye loaded in the NMOFs, resulting in the luminescence of the loaded dye without external excitation. The resulting CRET signals relate to the concentrations of the miRNAs or the genes and allow the sensitive analysis of miRNAs and genes. In addition, the DNA hairpin-functionalized dye-loaded NMOF sensing modules were further applied to develop amplified miRNA or gene CRET-based sensing platforms. The dye-loaded NMOFs were modified with hairpin probes that include in their loop domain the recognition sequences for miRNA-155 or miRNA-21 or the recognition sequences for the p53 or BRCA1 genes. Subjecting the hairpin-modified NMOFs to the respective miRNAs or genes, in the presence of two hairpins *H*_*i*_ and *H*_*j*_ that include in their stem regions caged G-quadruplex subunit domains, results in the analyte-triggered opening of the probe hairpin linked to the NMOFs, and the opened hairpin tethers induce the cross-opening of the hairpins *H*_*i*_ and *H*_*j*_ by the hybridization chain reaction, HCR, resulting in the assembly of G-quadruplex wires tethered to the NMOFs. The binding of hemin to the HCR-generated chains yields hemin/G-quadruplex DNAzyme wires that enhance, in the presence of luminol/H_2_O_2_, the CRET processes in the hybrid nanostructures. These amplification platforms lead to the amplified sensing of miRNAs and genes. By mixing the Fl- and Rh 6G-loaded hairpin-functionalized UiO NMOFs, the multiplexed CRET detection of miRNA-155, miRNA-21 and the p53 and BRCA1 genes is demonstrated.

## Abstract 2

Hemin/G-quadruplex DNAzyme-modified metal–organic framework nanoparticles act as functional hybrids for the catalyzed oxidation of luminol by H_2_O_2_, causing chemiluminescence and activation of chemiluminescence resonance energy transfer to the dye loads.

## Graphical abstract

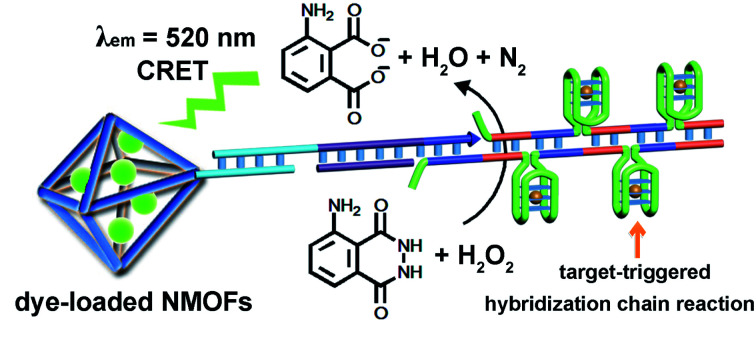

## Introduction

Substantial research efforts have been directed in the past two decades towards the development of sensors for the detection of DNA, RNA, microRNA (miRNA) and aptamer-based sensors.^1^ Numerous electrochemical,^2^ optical,^3^ microgravimetric^4^ and magnetic field-based sensors^5^ were developed, and ingenious amplification methods were demonstrated for the ultrasensitive detection of DNA-based sensors.^6^ Among the optical DNA-based sensors, fluorescence-based sensing platforms using FRET mechanisms,^7^ the application of luminescent quantum dots^8^ and luminescent Ag nanoclusters,^9^ and the use of DNAzyme-catalyzed chemiluminescent^10^ sensing platforms were demonstrated. In addition, optical plasmon-based DNA sensors involving absorbance changes associated with the aggregation of plasmonic particles and plasmonic coupling between nanoparticles^11^ and surface plasmon resonance transduction^12^ led to highly sensitive DNA sensing systems. Amplified DNA sensing schemes have included the application of DNA machineries, such as the replication/nicking machinery,^13^ rolling circle amplification,^14^ autocatalytic hybridization chain reaction (HCR),^15^ the biocatalytic regeneration of nucleic acid analytes in the presence of exonuclease,^16^ and so on. In addition, the development of multiplexed analysis schemes for the detection of genes or miRNAs has attracted continuous efforts. Different sized semiconductor quantum dots^17^ or silver nanoclusters,^18^ exhibiting size-controlled luminescence functions, and the selective desorption of fluorophores from graphene oxide supports^19^ were used to design multiplexed sensing assays. Also, different DNAzymes associated with DNA machineries or DNA nanostructures were used for multiplexed analysis of genes.^20^ In addition, the chemiluminescence resonance energy transfer (CRET) process was reported to be a useful path for the multiplexed analysis of genes.^21^ The hemin/G-quadruplex-modified semiconductor quantum dot hybrids act as useful functional structures for activation of the luminescence of the quantum dots through the chemiluminescence energy transfer path. The hemin/G-quadruplex-stimulated generation of chemiluminescence, upon the catalyzed oxidation of luminol by H_2_O_2_, provides a useful energy source for the excitation of the quantum dots with direct excitation. Accordingly, by the modification of different-sized semiconductor quantum dots functionalized with hairpin DNA probes that include target-specific sensing domains and caged G-quadruplex sequences, the multiplexed CRET analysis of different genes was demonstrated.^21^

The detection of miRNAs is of particular interest. miRNAs are short noncoding RNA sequences (19–26 bases) that control gene expression in various cellular transformations.^22^ The up-regulation or down-regulation of miRNAs has been related to biological processes, such as proliferation or apoptosis and various diseases. In particular, sequence-specific miRNAs are biomarkers for various types of cancer cell. Indeed, substantial research efforts have been directed to the development of sensing platforms for miRNAs.^23^ The low level of miRNAs in cancer cells requires, however, the development of analytical amplification methods and different pathways to detect miRNAs by exponential amplification using endonuclease,^24^ the application of the hybridization chain reaction (HCR)^25^ and catalytic hairpin assembly were demonstrated.^26^ Recent reports applied photoactivated toehold-mediated strand displacement and DNAzyme-driven nanomotors as amplification methods for analyzing miRNAs.^27^

Metal–organic framework nanoparticles, NMOFs, represent a class of porous materials^28^ that find broad applications in catalysis,^29^ storage and separation of gases,^30^ drug delivery carriers^31^ and sensing.^32^ Recently, the synthesis of Cu^2+^-bipyridine NMOFs, exhibiting horseradish peroxidase activities reflected by the generation of chemiluminescence upon the catalytic oxidation of luminol by H_2_O_2_, was reported.^33^ Upon the postmodification of bipyridine ligands with Cu^2+^ ions, the Cu^2+^-modified NMOFs yielded a functional module for inducing the CRET in the hybrid carriers and the generation of the fluorescein fluorescence without external excitation. Nonetheless, no sensing functions by these hybrid carriers were demonstrated. Modified NMOFs with signal-triggered reconfigurable nucleic acids have recently been introduced as stimuli-responsive drug carriers.^34^ Different triggers to unlock nucleic acid-gated NMOFs were discussed, including the pH-induced dissociation of i-motif or triplex nucleic acid gates,^35^ and the use of miRNAs^36^ or ligand–aptamer complexes^37^ to unlock nucleic acid-gated NMOFs were reported.

In the present study, we introduce dye-loaded UiO-66 NMOFs^38^ modified with hemin/G-quadruplex units as hybrid modules that act as functional nanostructures for the generation of CRET. We demonstrate the ON/OFF switchable generation of CRET by the hybrid nanoparticles. In addition, through the engineering of functional nucleic acid hairpin structures on the NMOFs, we introduce CRET-based miRNA and gene sensing platforms. By designing mixtures of appropriately engineered nucleic acid hairpin-modified dye-loaded NMOF hybrids, the multiplexed CRET analysis of miRNAs and genes is demonstrated. Furthermore, by coupling the HCR to the hairpin-modified dye-locked NMOF modules, the amplified CRET-based sensing of miRNAs or genes is achieved. The novelty of the study rests on the unique properties of hemin/G-quadruplex to catalyze the generation of chemiluminescence and the feasibility to confine, in the porous structure of UiO-66 NMOFs, a high loading of dyes in proximity to the hemin/G-quadruplex modifiers, a hybrid composite that allows effective CRET and multiplexed detection of the analytes. It should be noted that recently, hemin/G-quadruplex modified NMOFs were applied for the amplified chemiluminescent imaging of miRNA-133a in sera.^39^ In contrast to this study, our amplified hemin/G-quadruplex NMOF hybrid miRNA and gene sensing platforms apply the CRET mechanism as a readout signal, which allows the multiplexed analysis of different miRNAs or genes *via* the encapsulation of different CRET acceptor dyes in the NMOF carriers.

## Results and discussion, and experimental

**Fig. 1:**
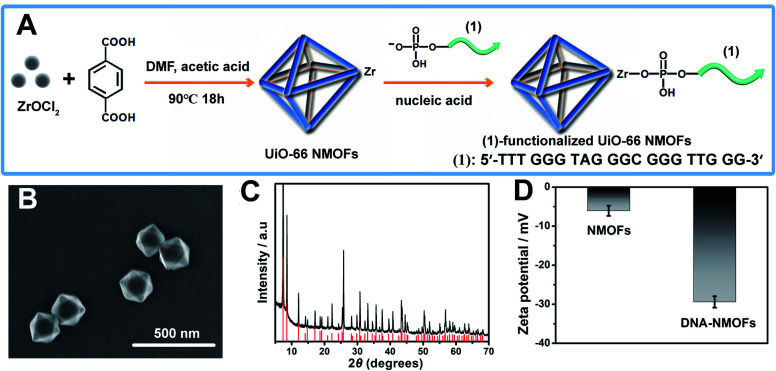
(A) Synthesis of the nucleic acid (**1**)-functionalized UiO-66 NMOFs. (B) SEM image of the (**1**)-modified UiO-66 NMOFs. (C) XRD pattern of the UiO-66 NMOFs (black), and simulated XRD pattern of the UiO-66 NMOFs (red). (D) Zeta-potentials corresponding to the bare UiO 66-NMOFs and the nucleic acid (**1**)-functionalized UiO-66 NMOFs. Error bars derived from *N* = 3 experiments.

Fig. 1(A) depicts the synthesis of the nucleic acid-modified UiO-66 metal–organic framework nanoparticles (NMOFs). The UiO-66 NMOFs were prepared by the reaction between ZrOCl_2_ and terephthalic acid. The 5′-end of the nucleic acid (**1**) was phosphorylated and was ligated to the vacant Zr^4+^-ion ligation sites on the NMOFs. Bipyramidal UiO-66 NMOFs were formed; Fig. 1(B) and dynamic light scattering experiments (Fig. S1†) indicate an average diameter of 200 nm for the nanoparticles. The crystallinity of the UiO-66 NMOFs was determined by powder X-ray diffraction (XRD, Fig. 1(C)). The zeta-potential of the NMOFs before and after modifying with (**1**) corresponded to −5 mV and −30 mV, respectively (Fig. 1(D)).

**Fig. 2:**
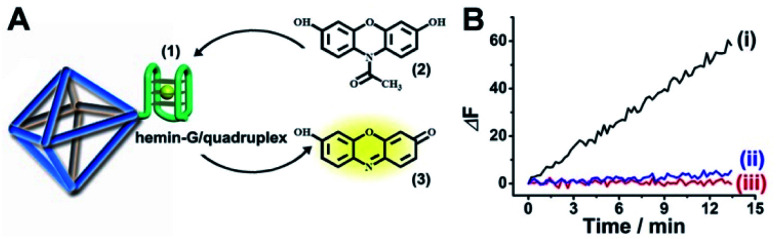
(A) Schematic hemin/G-quadruplex DNAzyme-modified UiO-66 NMOFs catalyzing the oxidation of Amplex Red (**2**) to the fluorescent resorufin (**3**). (B) Time-dependent fluorescence changes corresponding to the catalyzed oxidation of Amplex Red (**2**) to the fluorescent resorufin (**3**), in the presence of K^+^-ions, hemin and H_2_O_2_ (curve (i)). Control experiments showing the time-dependent fluorescence changes generated by the (**1**)-modified NMOFs in the presence of Amplex Red/H_2_O_2_ and in the absence of K^+^-ions and hemin (curve ii) and the hemin/G-quadruplex-modified NMOFs treated with CE in the presence of K^+^-ions, hemin and Amplex Red/H_2_O_2_ (curve iii).

The strand (**1**) is guanosine-rich. In the presence of K^+^-ions, the strands assemble into G-quadruplex units that associate with hemin and form hemin/G-quadruplex horseradish peroxidase-mimicking DNAzyme units.^40^ The DNAzyme units catalyze the oxidation of Amplex Red (**2**) by H_2_O_2_ to the fluorescent resorufin, (**3**) (Fig. 2(A)). Treatment of the hemin/G-quadruplex-modified NMOFs with 18-crown-6-ether (CE) that eliminates the K^+^-ions from the G-quadruplex *via* complexation with the CE^41^ results in the separation of the G-quadruplex, leading to catalytically inactive NMOFs. Fig. 2(B) curve (i) shows the K^+^-ions-stimulated formation of the active hemin/G-quadruplex DNAzyme structure that catalyzes the oxidation of Amplex Red to resorufin. Curve (ii) shows the catalytically inactive configuration of the (**1**)-functionalized NMOFs, and curve (iii) shows the reverse deactivation of the DNAzyme upon CE separation of the G-quadruplex. The switchable activation and deactivation of the hemin/G-quadruplex peroxidase activities could be switched to “ON” and “OFF” states for three cycles, in the presence of K^+^-ions/CE with no noticeable effect on the catalytic activity of the DNAzyme. It should be noted that the hemin/G-quadruplex-catalyzed oxidation of Amplex Red by H_2_O_2_ to the fluorescent resorufin was used to evaluate the loading of (**1**) on the UiO-66 NMOFs. Fig. S2(A)† shows the time-dependent rates of biocatalyzed oxidation of Amplex Red by H_2_O_2_ to resorufin in the presence of variable concentrations of the hemin/G-quadruplex DNAzyme, and Fig. S2(B)† shows the derived calibration curve. This calibration curve was used to evaluate the loading of the DNAzyme linked to a fixed amount of the NMOFs, Fig. S2(C),† and to derive the loading of the DNAzyme on the NMOFs that corresponds to 5 nmol of the G-quadruplex per mg NMOFs.

**Fig. 3:**
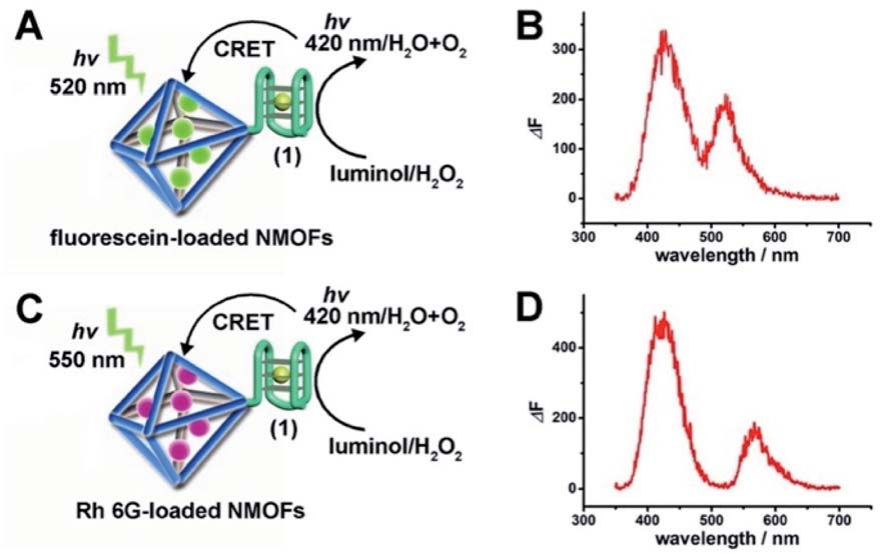
(A) Schematic CRET between the chemiluminescence generated by the hemin/G-quadruplex-catalyzed oxidation of luminol by H_2_O_2_ and the Fl-loaded UiO-66 NMOFs. (B) The spectrum of chemiluminescence generated by the hemin/G-quadruplex-catalyzed oxidation of luminol, *λ*_em_ = 420 nm, and the CRET luminescence signal of Fl at *λ*_em_ = 520 nm. (C) Schematic CRET between the chemiluminescence generated by the hemin/G-quadruplex-catalyzed oxidation of luminol by H_2_O_2_ and the Rh 6G-loaded UiO-66 NMOFs. (D) The spectrum of chemiluminescence generated by the hemin/G-quadruplex-catalyzed oxidation of luminol, *λ*_em_ = 420 nm, and the CRET luminescence signal of Rh 6G at *λ*_em_ = 550 nm.

The (**1**)-modified UiO-66 NMOFs were, then, loaded with fluorescein (Fl) or rhodamine 6G (Rh 6G). Since the unfolded (**1**) strand includes the guanosine (G)--rich sequence, the addition of K^+^-ions to the Fl- or Rh 6G-loaded NMOFs resulted in the self-assembly of K^+^-ions-stabilized G-quadruplex structures that acted as gates to lock the dyes in the NMOFs. After extensive washing of the NMOFs, the loading of the NMOFs with Fl and Rh 6G was evaluated to be 80 nmol mg^−1^ and 70 nmol mg^−1^ NMOFs, respectively (see Fig. S3 and S4† and accompanying discussion). The hemin was then incorporated into the G-quadruplex associated with the NMOFs. As shown in Fig. 3(A) and (C), the resulting hemin/G-quadruplex units catalyzed the generation of chemiluminescence through the catalyzed oxidation of luminol (*λ*_em_ = 420 nm), and the resulting chemiluminescence stimulated the chemiluminescence resonance energy transfer (CRET) to Fl or Rh 6G reflected by the CRET-stimulated emission of Fl (*λ*_em_ = 520 nm) or Rh 6G (*λ*_em_ = 550 nm) (Fig. 3(B) and (D)), respectively. It should be noted that dye-loaded NMOFs modified with single-stranded nucleic acids do not provide effectively locked porous structures, and the dye undergoes release (leakage) within several hours. Nonetheless, duplex, duplex-based hairpin or G-quadruplex gated NMOFs provide effective locks that prevent the leakage of the dye, due to steric closure of the pores. It should be noted that the dyes are locked in the pores separated by the octahedral subunits comprising the UiO-66 NMOFs and locked in the pores by the DNA locks, rather than being trapped in the octahedral subunits of the particles. Nonetheless, for the CRET process, the dyes have to be confined in the void volumes bridging the octahedral subunits of the NMOFs and for schematic presentation to preserve the dyes in confined nanoenvironments, we introduce them schematically into the octahedral subunits.

**Fig. 4:**
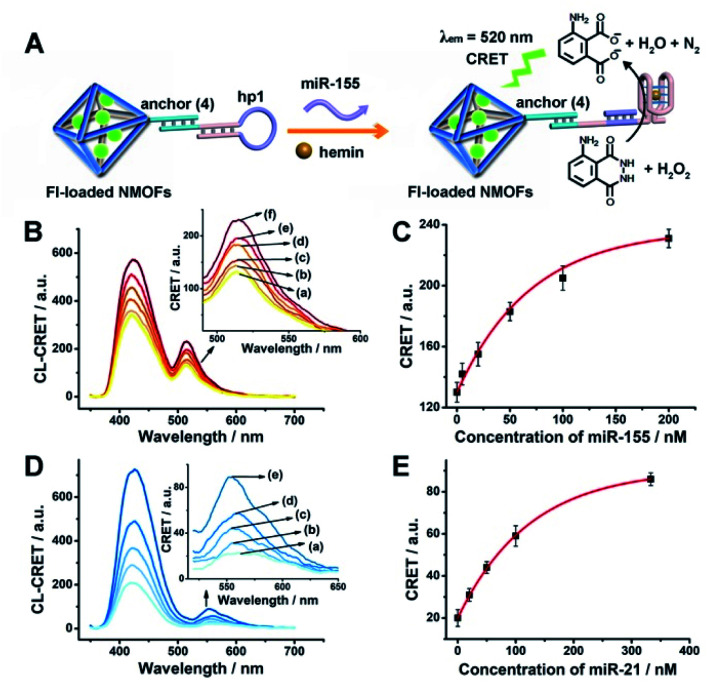
(A) Fl-loaded hairpin hp1-functionalized UiO-66 NMOFs for the CRET analysis of miRNA-155. The miRNA-155-triggered opening of the hairpin leads, in the presence of K^+^-ions and hemin, to the self-assembly of the hemin/G-quadruplex that catalyzes the generation of chemiluminescence, *λ*_em_ = 420 nm, and the CRET-induced fluorescence of Fl, *λ*_em_ = 520 nm. (B) CRET-induced fluorescence spectra of Fl, *λ*_em_ = 520 nm, in the presence of variable concentrations of miRNA-155: (a) 0 nM; (b) 5 nM; (c) 20 nM; (d) 50 nM; (e) 100 nM; (f) 200 nM. (C) Derived calibration curve corresponding to the CRET fluorescence signals of Fl generated at variable concentrations of miRNA-155. (D) CRET-induced fluorescence spectra of Rh 6G, *λ*_em_ = 550 nm, in the presence of variable concentrations of miRNA-21, according to Fig. S7:† (a) 0 nM; (b) 20 nM; (c) 50 nM; (d) 100 nM; (e) 333 nM. (E) Derived calibration curve corresponding to the CRET fluorescence signals of Rh 6G generated at variable concentrations of miRNA-21. Error bars derived from *N* = 3 experiments.

The successful hemin/G-quadruplex CRET-stimulated generation of fluorescence of the dyes encapsulated in the NMOFs was then used to develop CRET-based microRNA (miRNA) and gene sensing systems, and multiplexed sensing platforms. Specifically, we focused on the detection of miRNA-155, overexpressed in HepG2 liver cancer cells, and miRNA-21, overexpressed in MDA-MB-231 breast cancer cells. Fig. 4(A) shows the CRET-based sensing platform for miRNA-155. The UiO-66 NMOFs were functionalized with the phosphorylated tether (**4**) and loaded with Fl. The Fl-loaded (**4**)-modified NMOFs were hybridized with hairpin hp1 to lock the dye in the NMOFs. No leakage of the dye from the (**4**)/hp1-gated Fl-loaded NMOFs could be detected (see Fig. S5†). Hairpin hp1 was engineered to include in its loop domain the sequence recognizing miRNA-155. The G-quadruplex sequence was embedded, and caged, in the stem region of hairpin hp1. The caged structure of the G-quadruplex sequence prohibits the formation of the G-quadruplex module. In the presence of miRNA-155, the hp1 structure is opened, resulting in the uncaging of the stem domain, and in the presence of K^+^-ions and hemin, the hemin/G-quadruplex unit is formed on the surface of NMOFs. This leads to the hemin/G-quadruplex catalyzed oxidation of luminol by H_2_O_2_ and to the formation of chemiluminescence that stimulates CRET process to the embedded dye and the fluorescence of Fl (*λ*_em_ = 520 nm). Fig. 4(B) shows the chemiluminescence and CRET signals generated by the Fl-loaded UiO-66 NMOFs upon sensing different concentrations of miRNA-155. As the concentration of miRNA increases, the CRET signal is higher, consistent with the enhanced uncaging of the NMOFs and the increased content of the CRET-generating catalyst, the hemin/G-quadruplex. Fig. 4(C) shows the derived calibration curve. The system allowed the sensing of miRNA-155 with a detection limit that corresponds to 1.7 nM. (Detection limits in the study followed IUPAC guidelines;^42^ see the ESI.†) The sensing of miRNA-155 by the Fl-loaded NMOFs was selective. Treatment of the NMOFs with a foreign miRNA, such as miRNA-145, did not lead to any CRET signals (see Fig. S6†).

Using the same procedure, the (**4**)-modified NMOFs were loaded with Rh 6G, and the Rh 6G-loaded NMOFs were locked with a second hairpin (hp2), designed to detect miRNA-21 (for the schematic configuration of the miRNA-21-responsive NMOFs see Fig. S7, ESI†). Hairpin hp2 includes, in its loop region, the miRNA-21 recognition sequence, and in its stem region, the caged G-quadruplex inactive sequence. miRNA-21 is overexpressed in MDA-MB-231 breast cancer cells, and, thus, provides a biomarker for the detection of these cells. Thus, the Rh 6G-loaded (**4**)/hp2-gated NMOFs were applied to sense miRNA-21. The chemiluminescence spectra and CRET spectra generated by the DNAzyme-modified NMOFs are presented in Fig. 4(D) and the derived calibration curve is shown in Fig. 4(E). The NMOFs enabled the analysis of miRNA-21 with a detection limit that corresponded to 6.7 nM.

**Fig. 5:**
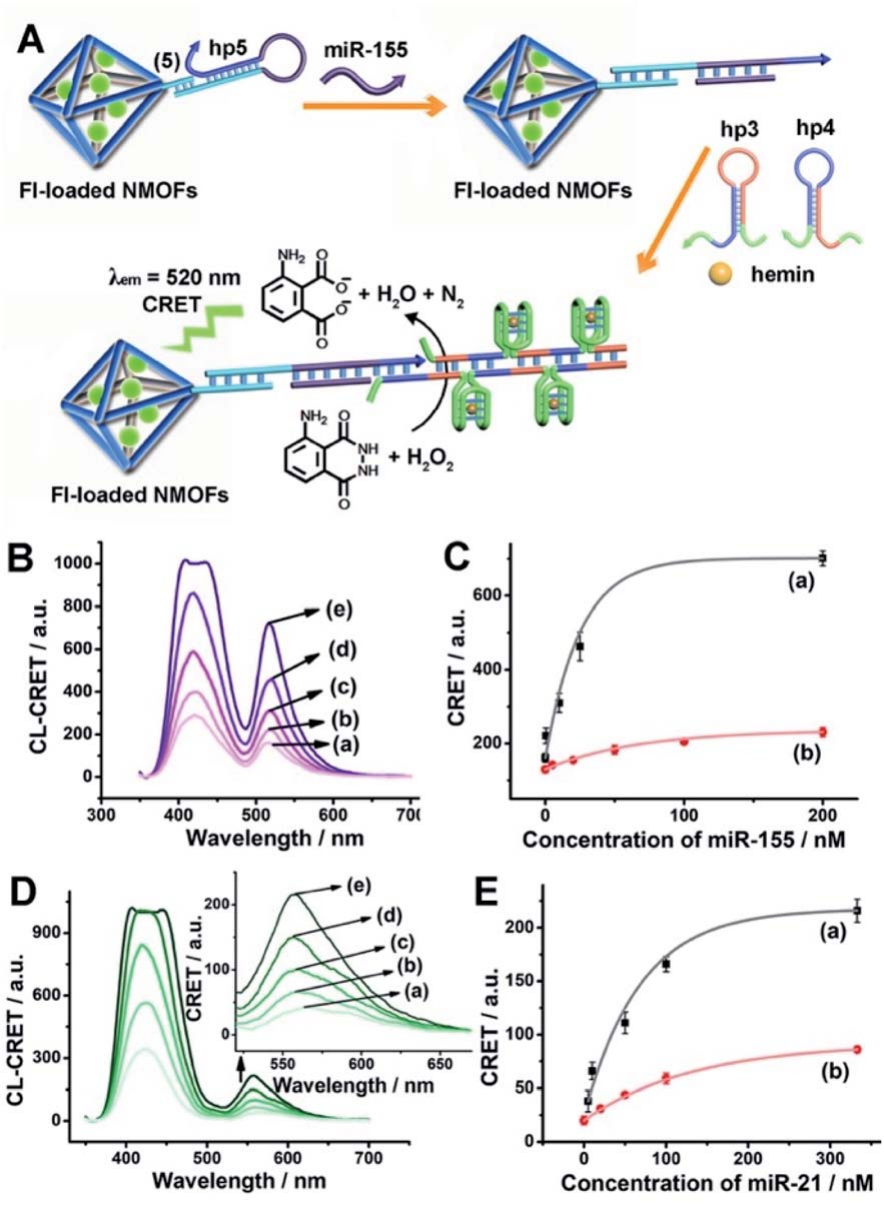
(A) Amplified CRET-induced detection of miRNA-155 by coupling the HCR generating hemin/G-quadruplex polymers to the Fl-loaded NMOFs. (B) Chemiluminescence and CRET-generated fluorescence of Fl upon analyzing various concentrations of miRNA-155 by the scheme outlined in (A): (a) 0 nM; (b) 0.5 nM; (c) 10 nM; (d) 25 nM; (e) 200 nM. (C) Calibration curve corresponding to the CRET-generated fluorescence of Fl in the presence of variable concentrations of miRNA-155: (a) using the HCR-amplified CRET-detection scheme shown in (A); (b) using the non-amplified hairpin-functionalized NMOFs according to Fig. 4(A). (D) Chemiluminescence and CRET-generated fluorescence of Rh 6G upon analyzing various concentrations of miRNA-21 according to the scheme in Fig. S8:† (a) 0 nM; (b) 5 nM; (c) 50 nM; (d) 100 nM; (e) 333 nM. (E) Calibration curve corresponding to the CRET-generated fluorescence of Rh 6G in the presence of variable concentrations of miRNA-21: (a) using the HCR-amplified CRET-detection scheme shown in Fig. S8;† (b) using the non-amplified hairpin-functionalized NMOFs according to Fig. 4(E). Error bars derived from *N* = 3 experiments.

The low concentrations of miRNAs in biological samples require, however, the development of amplification paths for the analysis of miRNAs. Different methods to amplify miRNA detection platforms were reported, and these included the regeneration of the miRNAs using exonuclease III, Exo III, and a rolling circle amplification process. As the CRET signal is controlled by the concentration of the hemin/G-quadruplex catalyst units, we argued that the increase of the hemin/G-quadruplex generated by a single opening event of the hairpin probes could enhance the CRET signal, and thereby, improve the sensitivity of the sensing platform. Accordingly, we applied the hybridization chain reaction (HCR) as a path to amplify the miRNA detection platform (Fig. 5(A)). The hairpin, hp 5, was linked to the Fl-loaded anchor (**5**)-modified UiO-66 NMOFs. The loop of hp5 was engineered to include the miRNA-155 recognition sequence. In the presence of miRNA-155, the hairpin is opened, leading to a free tether that triggers, in the presence of hairpins hp3 and hp4, the HCR process, where the cross-opening of hp3 and hp4 leads to the oligomers of self-assembled G-quadruplex chains that catalyze the generation of chemiluminescence, and to the subsequent process of CRET to the Fl dye-loaded NMOFs carriers, leading to the fluorescence of Fl (*λ*_em_ = 520 nm). The HCR-stimulated formation of multiple hemin/G-quadruplex catalytic units amplifies the resulting CRET signal. Fig. 5(B) shows the chemiluminescence and CRET-induced Fl fluorescence spectra generated by the NMOFs at different concentrations of miRNA-155. The resulting calibration curve is shown in Fig. 5(C) (curve (a)). For comparison, in Fig. 5(C), curve (b) shows the calibration curve corresponding to the CRET analysis of miRNA-155 by the single hemin/G-quadruplex-labeled Fl-loaded NMOFs, *e.g.*Fig. 4 (similar content of Fl-loaded NMOFs). Evidently, the CRET signals for sensing miRNA-155 are significantly amplified by the HCR amplification path. The HCR-amplified generation of the Cr CRET signal allowed the analysis of miRNA-155 with a detection limit corresponding to 0.17 nM. Thus, the application of the HCR amplification path allowed a 10-fold improvement in the detection limit of miRNA-155.

Using the same approach, the Rh 6G-loaded NMOFs were modified with hairpin hp6, which was engineered to amplify the sensing of miRNA-21, through the HCR process, and the accompanying CRET signal (fluorescence of Rh 6G at *λ* = 550 nm) (Fig. S8†). The resulting chemiluminescence and Rh 6G chemiluminescence resonance fluorescence spectra at different concentrations of miRNA-21 and the resulting calibration curve are shown in Fig. 5(E) curve (a). The system allowed the analysis of miRNA-21 with a detection limit of 1.67 nM, a sensitivity value that is *ca.* 4-fold higher as compared to the detection limit demonstrated by the single hemin/G-quadruplex functionalized NMOFs (Fig. 5(E), curve (b)).

**Fig. 6:**
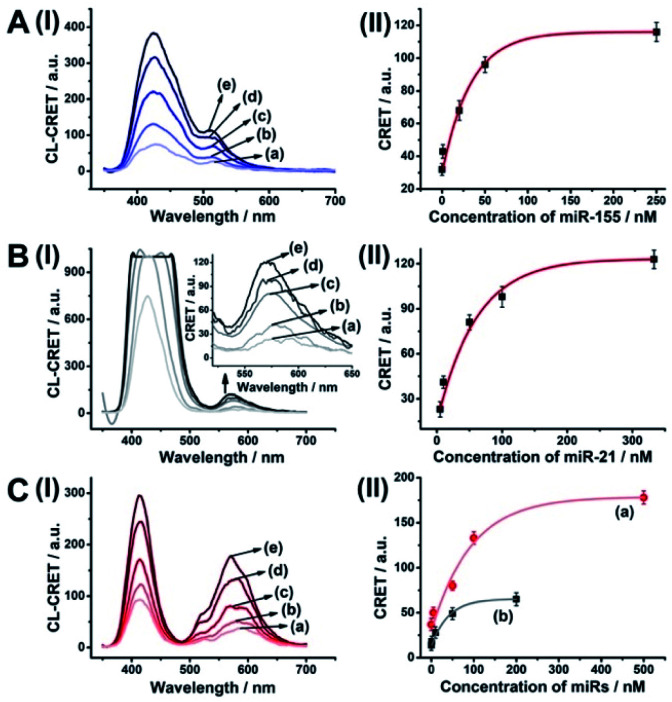
Multiplexed analysis of miRNA-155 and miRNA-21 by a mixture consisting of the Fl-loaded hp5-modified NMOFs and the Rh 6G-loaded hp6-modified NMOFs using the HCR-amplified CRET-induced analysis of the two miRNAs upon (panel A) subjecting the mixture to variable concentrations of miRNA-155: (a) 0 nM; (b) 1 nM; (c) 20 nM; (d) 50 nM; (e) 250 nM; (panel B) subjecting the mixture to variable concentrations of BRCA1: (a) 0 nM; (b) 5 nM; (c) 50 nM; (d) 100 nM; (e) 333 nM; and (panel C) subjecting the mixture to variable concentrations of miRNA-155: (a) 0 nM; (b) 1 nM; (c) 10 nM; (d) 50 nM; (e) 200 nM (curve (b) in panel II), and to variable concentrations of miRNA-21: (a) 0 nM; (b) 5 nM; (c) 50 nM; (d) 100 nM; (e) 500 nM (curve (a) in panel II). Error bars derived from *N* = 3 experiments.

The HCR amplified sensing of miRNA-155 or miRNA-21 was then applied for the multiplexed analysis of the two miRNAs (Fig. 6). A mixture of Fl-loaded miRNA-155-responsive NMOFs and Rh 6G-loaded miRNA-21-responsive NMOFs was exposed to miRNA-155 and/or miRNA-21. In the presence of miRNA-155, only the HCR process associated with the hp5-functionalized Fl-loaded NMOFs is activated, leading to the CRET signal of Fl (Fig. 6(A), panel I and panel II). In the presence of miRNA-21, only the HCR process associated with the hp6-modified Rh 6G-loaded NMOFs is activated, generating the CRET-induced signal of Rh 6G (Fig. 6(B), panel I and panel II). In the presence of miRNA-155 and miRNA-21, the HCR process associated with the two kinds of NMOF is activated, leading to the CRET-stimulated fluorescence of Fl and Rh 6G (Fig. 6(C), panel I and panel II). Note that due to the different CRET signal intensities of the fluorophores, the mixture of Fl/Rh 6G-loaded NMOFs consisted of a ratio of 1 : 3 to obtain a clear separation of the CRET signals generated by the two fluorophores. (For the inefficient multiplexed analysis of miRNA-155 and miRNA-21 by the non-amplified mixture of NMOFs described in Fig. 4 and S7, see ESI Fig. S9† and accompanying discussion.)

**Fig. 7:**
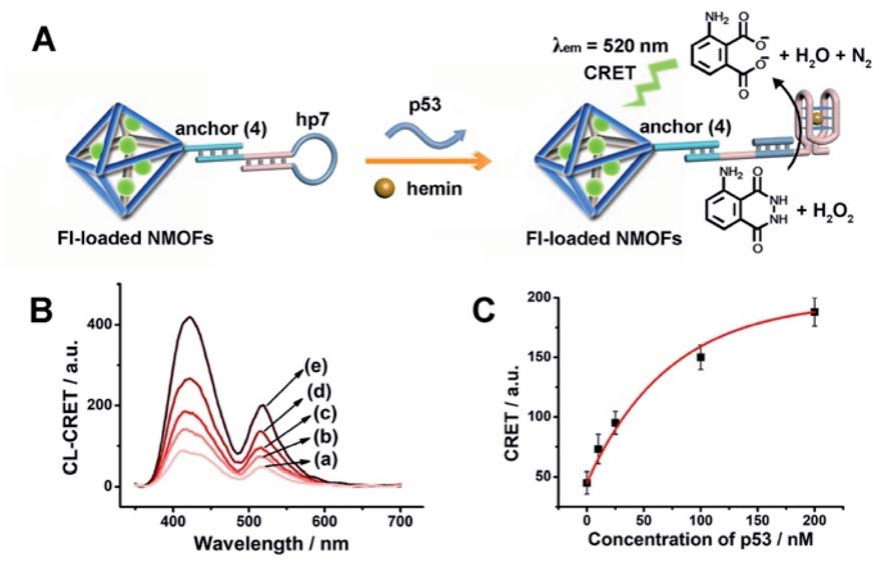
(A) Fl-loaded hairpin hp7-functionalized UiO-66 NMOFs for the CRET analysis of gene p53. The p53-triggered opening of the hairpin leads, in the presence of K^+^-ions and hemin, to the self-assembly of the hemin/G-quadruplex that catalyzes the generation of chemiluminescence, *λ*_em_ = 420 nm, and the CRET-induced fluorescence of Fl, *λ*_em_ = 520 nm. (B) CRET-induced fluorescence spectra of Fl, *λ*_em_ = 520 nm, in the presence of variable concentrations of p53: (a) 0 nM; (b) 10 nM; (c) 25 nM; (d) 100 nM; (e) 200 nM. (C) Derived calibration curve corresponding to the CRET fluorescence signals of Fl generated at variable concentrations of p53. Error bars derived from *N* = 3 experiments.

The CRET-based analysis of nucleic acids by hemin/G-quadruplex-modified dye-loaded NMOFs was further applied for the detection of genes and for the multiplexed analysis of the p53 and BRCA1 genes. Fig. 7(A) depicts the sensing platform for the analysis of the p53 gene. The anchor (**4**)-modified UiO-66 NMOFs were loaded with Fl, and hairpin hp7 was hybridized with the (**4**)-anchor units associated with the NMOFs. The loop domain of hp7 included the recognition sequence for p53, and the stem region of the hairpin included the G-quadruplex sequence in a caged configuration. In the presence of the p53 gene, hairpin hp7 is opened, leading to the uncaged G-quadruplex sequence, and to the formation of the hemin/G-quadruplex units. The resulting DNAzyme catalyzed oxidation of luminol by H_2_O_2_ generated chemiluminescence and the CRET-guided fluorescence of Fl (*λ*_em_ = 520 nm) (Fig. 7(B)). The resulting signal is controlled by the concentrations of analyte p53, and as the concentration of p53 increases, the CRET signals are intensified. The sensing platform enabled the detection of p53 with a detection limit corresponding to 3.3 nM (Fig. 7(C)). Similarly, the hairpin hp8 was hybridized with the (**4**)-modified Rh 6G-loaded NMOFs (Fig. S10(A)†). The loop domain of hp8 included the recognition sequence of the BRCA1 gene, while the stem part included the caged G-quadruplex sequence. In the presence of the BRCA1 gene, hairpin hp8 was opened, resulting in the formation of the catalytic hemin/G-quadruplex. In the presence of H_2_O_2_ and luminol, the hemin/G-quadruplex catalyzed the generation of chemiluminescence, and the CRET-guided fluorescence of Rh 6G (*λ*_em_ = 550 nm) proceeded (Fig. S10 (B)†). The CRET signals were controlled by the concentrations of the BRCA1 gene, and as they increased, the CRET signals were intensified (Fig. S10(C)†). The method enabled the analysis of the BRCA1 gene with the detection limit corresponding to 6.7 nM. (For the multiplexed analysis of p53 and/or BRCA1 by the mixture consisting of the Fl-loaded hp7-functionalized NMOFs and the Rh 6G-loaded hp8-functionalized NMOFs, see ESI Fig. S11† and accompanying discussion.)

**Fig. 8:**
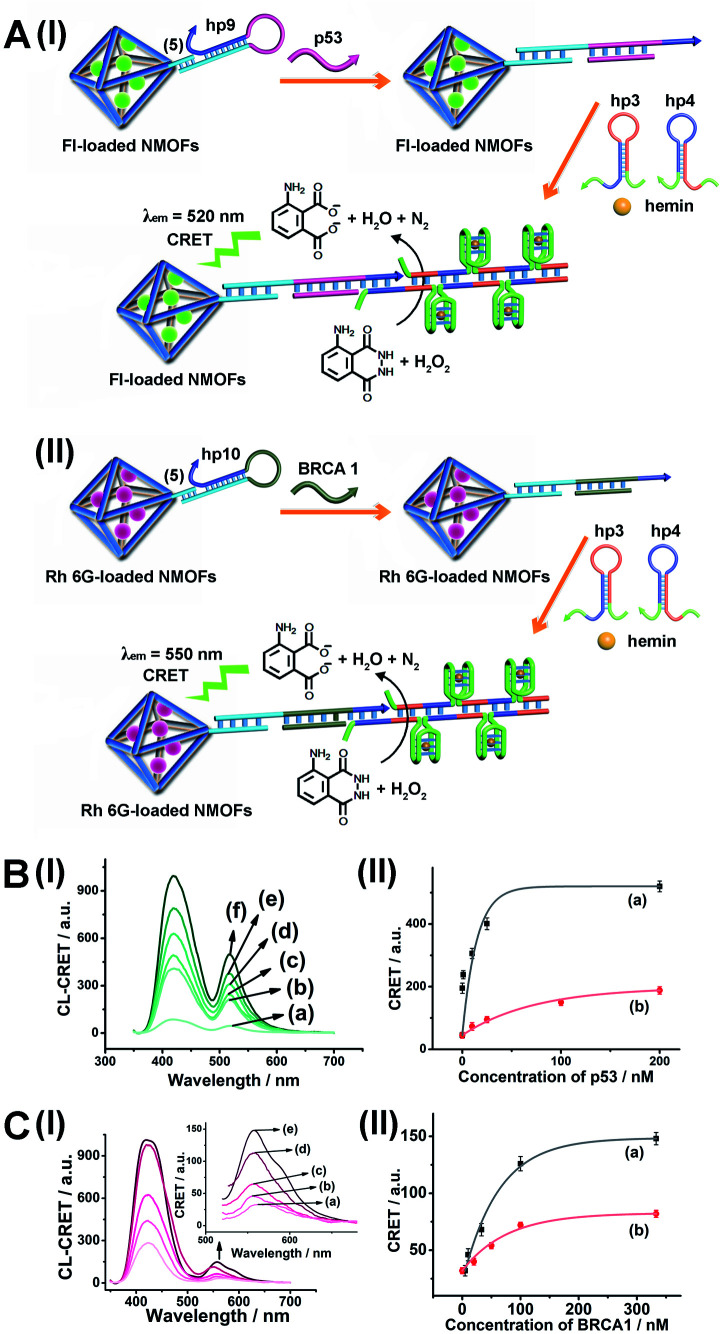
(A) Panel I: amplified CRET-induced detection of p53 by coupling the HCR generating hemin/G-quadruplex polymers to the Fl-loaded NMOFs. Panel II: amplified CRET-induced detection of BRCA1 by coupling the HCR generating hemin/G-quadruplex polymers to the Rh 6G-loaded NMOFs. (B) Panel I: chemiluminescence and CRET-generated fluorescence of Fl upon analyzing various concentrations of p53 by the scheme outlined in (A) panel I: (a) 0 nM; (b) 0.5 nM; (c) 1 nM; (d) 10 nM; (e) 25 nM; (f) 333 nM. Panel II: calibration curve corresponding to the CRET-generated fluorescence of Fl in the presence of variable concentrations of p53. (C) Panel I: chemiluminescence and CRET-generated fluorescence of Rh 6G upon analyzing various concentrations of BRCA1 by the scheme outlined in (A) panel II: (a) 0 nM; (b) 5 nM; (c) 33 nM; (d) 100 nM; (e) 333 nM. Panel II: calibration curve corresponding to the CRET-generated fluorescence of Rh 6G in the presence of variable concentrations of BRCA1. Error bars derived from *N* = 3 experiments.

As before, we used the HCR method to amplify the CRET detection platform of the genes, and we applied the amplification method for the multiplexed analysis of the genes (Fig. 8(A), panel I and panel II). Hairpins hp9 and hp10 were hybridized, as gating units, with the (**5**)-modified Fl/Rh 6G-loaded NMOFs, respectively. The loop domains of hp9 and hp10 included the specific recognition sequences for the p53 or BRCA1 gene, respectively. In the presence of the p53 or BRCA1 genes, the respective hairpins are unlocked to initiate, in the presence of hairpins hp3 and hp4, the HCR process. Hairpins hp3 and hp4 were engineered to yield, upon the HCR process, the hemin/G-quadruplex units embedded in HCR chains that, in the presence of hemin/H_2_O_2_/luminol, improve the process of CRET to the fluorophores entrapped in the NMOF carriers, thereby increasing the sensitivities of the detection platforms. Fig. 8(B) panel I shows the chemiluminescence and CRET spectra generated by the Fl-loaded NMOFs, upon sensing the p53 gene, and Fig. 8(C), panel I shows the CRET spectra generated by the Rh 6G-loaded NMOFs, upon sensing the BRACA1 gene, using the HCR-amplified detection scheme. Fig. 8(B) and (C), panels II show the CRET signal-derived calibration curves corresponding to the analysis of p53 and BRCA1 genes by the Fl- and Rh 6G-loaded NMOFs. The p53 and BRCA1 genes are analyzed with detection limits corresponding to 0.33 nM and 1.67 nM, using the HCR amplified pathway. The sensitivities for sensing p53 and BRCA1 are 10-fold and 4-fold improved by the HCR amplification as compared to non-amplified sensing platform presented in Fig. 7 and S10.†

**Fig. 9:**
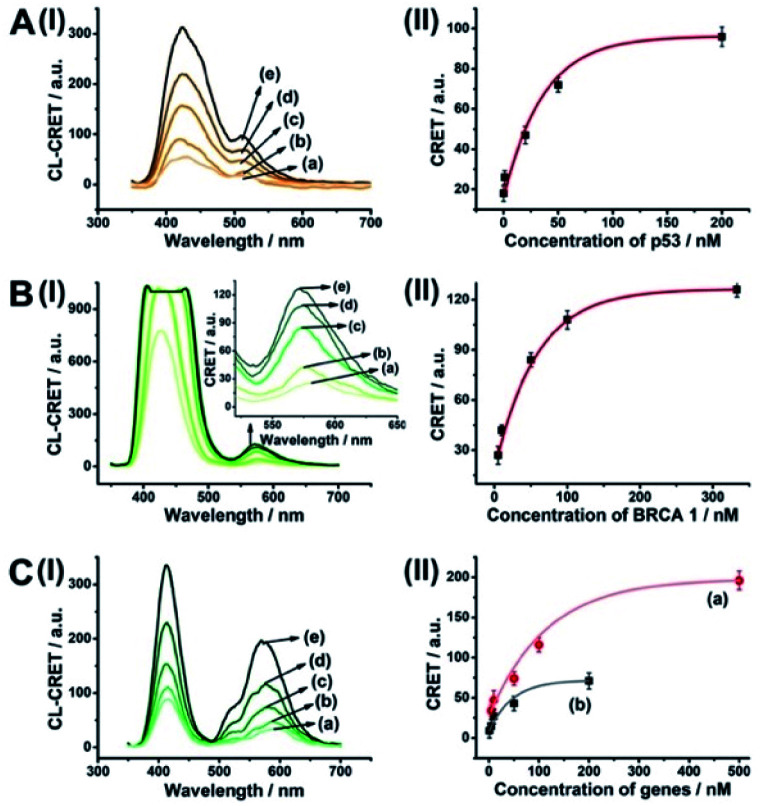
Multiplexed analysis of the p53 and BRCA1 genes by a mixture consisting of Fl-loaded hp9-modified NMOFs and Rh 6G-loaded hp10-modified NMOFs using the HCR-amplified CRET-induced analysis of the two genes upon (panel A) subjecting the mixture to variable concentrations of p53: (a) 0 nM; (b) 1 nM; (c) 20 nM; (d) 50 nM; (e) 200 nM; (panel B) subjecting the mixture to variable concentrations of BRCA1: (a) 0 nM; (b) 5 nM; (c) 50 nM; (d) 100 nM; (e) 333 nM; and (panel C) subjecting the mixture to variable concentrations of p53: (a) 0 nM; (b) 1 nM; (c) 10 nM; (d) 50 nM; (e) 200 nM (curve (b) in panel II), and to variable concentrations of BRCA1: (a) 0 nM; (b) 5 nM; (c) 50 nM; (d) 100 nM; (e) 500 nM (curve (a) in panel II). Error bars derived from *N* = 3 experiments.

As before, the HCR amplified CRET-detection platform can be used for the multiplexed analysis of the genes (Fig. S9†). The mixture of hp9-functionalized Fl-loaded NMOFs and hp10-modified Rh 6G-loaded NMOFs was used for the multiplexed analysis of the genes. In the presence of the p53 gene, only the HCR process associated with Fl-loaded NMOFs is activated, leading to the CRET spectra of Fl (Fig. 9(A), panel I and panel II). In the presence of the BRCA1 gene, only the HCR process associated with Rh 6G-loaded NMOFs is activated, leading to the CRET spectra of Rh 6G (Fig. 9(B), panel I and panel II). In the presence of the two genes p53 and BRCA1, the HCR process associated with the two kinds of NMOF is activated, leading to the CRET-stimulated signals corresponding to Fl (*λ*_em_ = 520 nm) and Rh 6G (*λ*_em_ = 550 nm) (Fig. 9(C), panel I and panel II).

## Conclusions

The present study has introduced metal–organic framework porous nanoparticles, NMOFs, as functional carriers of fluorophores for the chemiluminescence resonance energy transfer (CRET)-stimulated analysis of miRNAs and genes. The integration of hairpin units that self-assemble into functional hemin/G-quadruplex catalytic modules stimulated the generation of CRET-induced fluorescence outputs of the fluorophores entrapped in the NMOFs, upon sensing the miRNAs or the genes. By the integration of different fluorophores (energy acceptors) in the NMOFs, the multiplexed analysis of two different miRNAs or different genes was achieved. Furthermore, by the appropriate tailoring of the hairpins, caging the NMOFs, the amplified sensing of miRNAs or genes was demonstrated by coupling the hybridization chain reaction (HCR) to the sensing events occurring on the NMOFs. The CRET-induced sensing of analytes by the hairpin-functionalized NMOFs can be extended to other analytical targets, *e.g.* the detection of aptamer–ligand complexes. Beyond the significance of the fluorophore-loaded hairpin-functionalized NMOFs for sensing, the results are important because they introduce the concept of modifying and caging NMOFs by hairpin-responsive gates. Such functional NMOFs would be of significance for the triggered release of loads (drugs) entrapped in the NMOFs.

## Conflicts of interest

There are no conflicts to declare.

## Supplementary Material

SC-012-D0SC06744J-s001
