# Supplementary material for: Multiplexed and amplified chemiluminescence resonance energy transfer (CRET) detection of genes and microRNAs using dye-loaded hemin/G-quadruplex-modified UiO-66 metal–organic framework nanoparticles
Source: Chem Sci. 2021 Feb 8;12(13):4810–8. doi: 10.1039/d0sc06744j (PMC8179566; doi:10.1039/d0sc06744j)
Supplement: SC-012-D0SC06744J-s001 [file SC-012-D0SC06744J-s001.pdf]

Electronic Supplementary Material (ESI) for Chemical Science.
This journal is © The Royal Society of Chemistry 2021

Supporting Information

Multiplexed and Amplified Chemiluminescence Resonance Energy Transfer (CRET) Detection of Genes and microRNAs Using Dye-Loaded Hemin/G-Quadruplex-Modified UiO-66 Metal-Organic Framework Nanoparticles

*Pu Zhang, Yu Ouyang, and Itamar Willner**

Institute of Chemistry, Center for Nanoscience and Nanotechnology, The Hebrew University of Jerusalem, Jerusalem 91904, Israel.

E-mail: itamar.willner@mail.huji.ac.il

EXPERIMENTAL SECTION

Materials and Instruments. 2-[4-(2-Hydroxyethyl)piperazin-1-yl]ethanesulfonic acid sodium salt (HEPES), sodium hydroxide, magnesium chloride, fluorescein (Fl), Rhodamine 6G (Rh 6G), N, N, N', N'-tetramethy-lethylenediamine (TEMED), dimethylformamide (DMF), luminol, zirconium oxychloride ($ZrOCl_2$) and 2-aminoterephthalic acid were bought from Sigma-Aldrich. DNA oligonucleotides were synthesized and purified at Integrated DNA Technologies Inc. (Coralville, IA). Ultrapure water from NANOpure Diamond (Barnstead) source was applied throughout the whole experiments.

A Magellan XHR 400L scanning electron microscope (SEM) was employed to characterize the microcarriers. Fluorescence spectra was measured with a Cary Eclipse Fluorometer (Varian Inc.). The emission of the chemiluminescence measurements was measured from 350 nm to 700 nm. The concentrations of DNA oligonucleotides were monitored by UV-2401PC (SHIMADAZU).

The sequences of all nucleic acid used in this paper is listed as follows: (from 5' to 3')

Anchor **(1)**: PHOS-G4: PHOS-TTT GGG TAG GGC GGG TTG GG

miRNA-155: UUAAUGCUAAUCGUGAUAGGGGU

miRNA-21: UAGCUUAUCAGACUGAUGUUGA

mutant p53: TCATCACACTGGAAGACTC

BRCA1:AAAGTGTTTTTCATAAACCCATTATCCAGGACTGTTTATAGCTGTTGGAA
G

Single G/quadruplex uint-based sensing

Anchor **(4)**:Phos-TTTTTTTGTTT

Hp1:GGGTTGGGCGGGATGGGTTACCCCTATCACGATTAGCGGTAACCCAAAACA
AAAAAA

Hp2:GGGTTGGGCGGGATGGGTTTCAACATCAGTCTGATAAAAACCCAAAACAAA

AAAA

Hp7:GGGTTGGGCGGGATGGGTTGAGTCTTCCAGTGTGATGAAACCCAAAACAAA

AAA

HP8:GGGTTGGGCGGGATGGGTTCCAACAGCTATAAACAGTAATAACCCAAAACA

AAAAAA

hybridization chain reaction (HCR)-based sensing

Anchor **(5)**:TTTGTTTTTTT-Phos

HP3:AGGGCGGGTGGGTGTTTAAGTTGGAGAATTGTACTTAAACACCTTCTTCTTG

GGT

HP4:TGGGTCAATTCTCCAACTTAAACTAGAAGAAGGTGTTTAAGTTGGGTAGGGC

GGG

Hp5:AAAAAAACAAATTAAACACCTTACCCCTATCACGATTAGCATTAAAGAAGA

AGGTGTTTAAGTA

Hp6:AAAAAAACAAATTAAACACCTTTCAACATCAGTCTGATAAGCTAAGAAGAA

GGTGTTTAAGTA

Hp9: AAAAAAACAAATTAAACACCTTGAGTCTTCCAGTGTGATGAAGAAGAAGGT

GTTTAAGTA

HP10:AAAAAAACAAATTAAACACCTTCTTCCAACAGCTATAAACAGTCCTGGATA

ATGGGTTTATGAAAAACACTTTAGAAGAAGGTGTTTAAGTA

Synthesis of NMOFs. The preparation of NMOFs was according to the reported paper. First of all, 50 mg of as prepared terephthalic acid and 21 mg of $ZrOCl_2$ were mixed together in DMF (4 mL). Then, 2 mL of acetic acid was treated with the mixture in an oven and was reacted at 90 °C for 18 hours. After that, the as-prepared UiO-66 NMOFs were centrifuged and washed with DMF, triethylamine/ethanol (1:20, V/V), and ethanol for twice, respectively.

Synthesis of DNA-gated dye-loaded NMOFs. Initially, NMOF nanoparticles (1 mg, 1 mL) were treated with phosphorylated-DNA (100 nmol) and incubated over 12h. Then, the above solution was diluted with NaCl to a final concentration of 1 M and then the DNA-functionalized NMOFs were washed in water to remove unbounded nucleic acids. Next, 1 mg of NMOFs were treated with Rh 6G (0.5 mg·mL^{-1}) or Fl (0.5 mg·mL^{-1}) over 12 h in 1 mL water. The NMOFs were then transferred to a buffer solution and hybridized with corresponding DNA hairpins, resulting to the locked state of the NMOFs to encapsulate the drugs/dyes. Over 12 hours, the NMOFs were washed several times to remove the unloaded dyes. The dyes-loaded DNA-gated NMOFs were keep in 4 ^{o}C for further use.

The analysis of miRNAs/genes by the single G/quadruplex unit-based dye-loaded NMOFs using CRET as the readout signal. Take the analysis of miRNA-155 as example. The **(4)**/hp1-gated Fl-loaded NMOFs, 1 mg, was diluted in 25 mM HEPES, 20 mM Mg^{2+} and 200 mM Na^{+}, and was incubated with different concentrations of analyte miRNA-155, 200 mM K^{+} and 0.5 μM hemin for 1 h. Measurement was performed after addition of 0.75 mM luminol, 2.9 mM H_2O_2 and 0.01 M NaOH. The light emission intensity was measured immediately.

The analysis of miRNAs/genes by the HCR-based dye-loaded NMOFs using CRET as the readout signal. Take the analysis of miRNA-155 as example. The **(5)**/hp5-gated Fl-loaded NMOFs, 1 mg, was diluted in 25 mM HEPES, 20 mM Mg^{2+} and 200 mM Na^{+}, and was incubated with different concentrations of analyte miRNA-155, 1 μM of hp3 and hp4, 200 mM K^{+}, and 5 μM hemin for 1 h. Measurement was performed after addition of 0.75 mM luminol, 2.9 mM H_2O_2 and 0.01 M NaOH. The light emission intensity was measured immediately.

Evaluation of detection limits. The detection limits for the different sensing platforms followed IUPAC guidelines,[S1-2] using the equation DL=3S_B/m, where m is the slope of the calibration curve and S_B is the standard deviation of the blank.

References

S1. G. L. Long, and J. D. Winefordner, *Anal. Chem.,* 1983, **55**, 12A-14A.

S2. A-E, Radi, J. L. A. Sanchez, E. Baldrich, and C. K. O'Sullivan, *J. Am. Chem. Soc.,* 2006, **128**, 117-124.

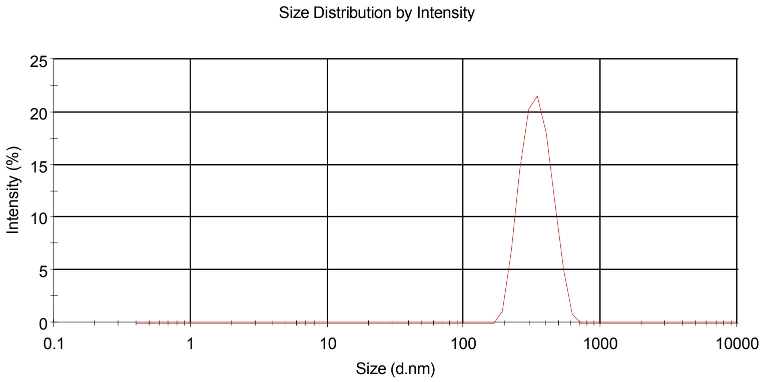

Figure S1. Dynamic light scattering data for UiO-66 NMOFs suspended in H_2O.

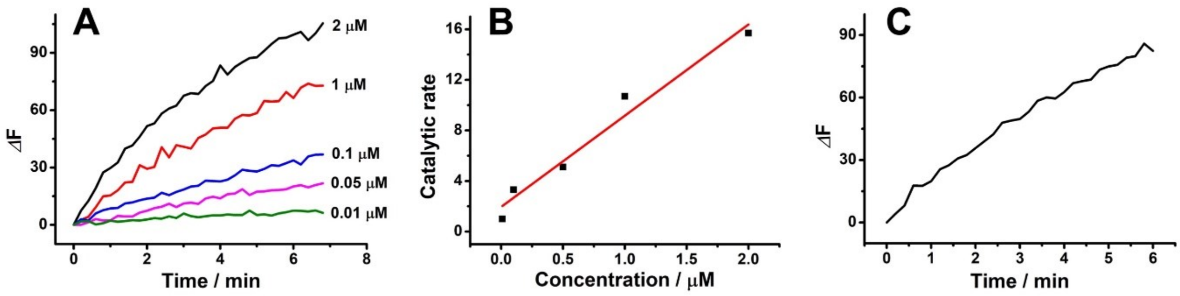

Figure S2. (A) Time-dependent fluorescence changes as a function of time in the presence of variable concentrations of the hemin/G-quadruplex DNAzyme. (B) The calibration curve corresponding to the fluorescence intensities as a function of the concentration of DNAzyme derived from (A). (C) Time-dependent fluorescence changes generated by DNAzyme-modified NMOFs. 0.04 mg of **(1)**-functionalized NMOFs were introduced in 0.1mL buffer solution containing 200 mM K^+, 0.2 mM Amplex Red, 833 μM H_2O_2 and 0.5 μM hemin. The fluorescence spectrum of the NMOFs solution was recorded, Figure S2 (C), and using the calibration curve shown in Figure S2 (B), the loading of DNAzyme corresponded to 5 nmols per 1 mg of NMOFs.

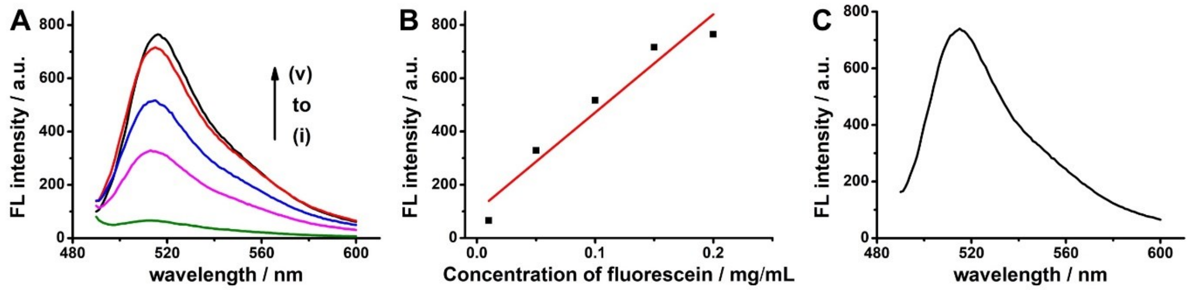

Figure S3. (A) Fluorescence intensities of the Fl dye with concentrations of (i) 0.01 mg/mL, (ii) 0.05 mg/mL, (iii) 0.1 mg/mL, (iv) 0.15 mg/mL, (v) 0.2 mg/mL. (B) The calibration curve corresponding to the fluorescence intensities as a function of the concentration of Fl derived from (A). (C) Evaluation of the loading of Fl on the NMOFs. 1 mg of NMOFs were introduced in 1mL of 0.2mg/mL Fl solution. The mixture was stirred for 12 hours. Afterwards, the NMOFs were precipitated and the fluorescence spectrum of the supernatant solution was recorded, Figure S3 (C), and using the calibration curve shown in Figure S3 (B), the loading of Fl corresponded to 80 nmols per 1mg of NMOFs.

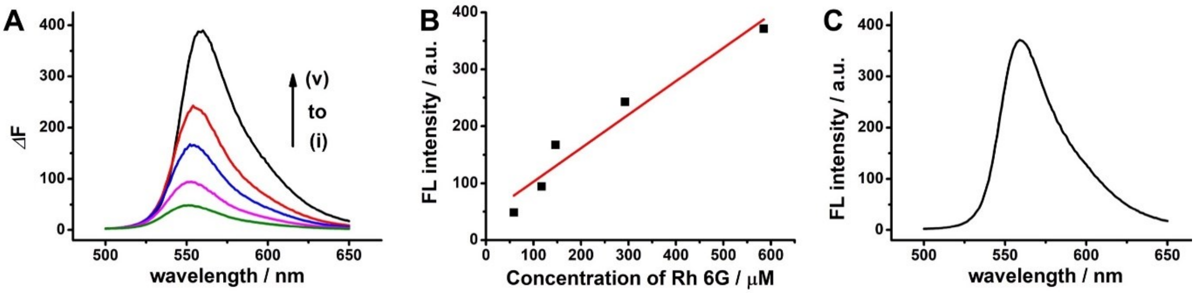

Figure S4. (A) Fluorescence intensities of the Rh 6G with concentrations of (i) 59 μM, (ii) 117 μM, (iii) 146 μM, (iv) 292 μM, (v) 585 μM. (B) The calibration curve corresponding to the fluorescence intensities as a function of the concentration of Rh 6G derived from (A). (C) Evaluation of the loading of Rh 6G on the NMOFs. 1 mg of NMOFs were introduced in 1mL of 0.57 mM Rh 6G solution. The mixture was stirred for 12 hours. Afterwards, the NMOFs were precipitated and the fluorescence spectrum of the supernatant solution was recorded,

Figure S4 (C), and using the calibration curve shown in Figure S4 (B), the loading of Rh 6G corresponded to 70 nmols per 1mg of NMOFs.

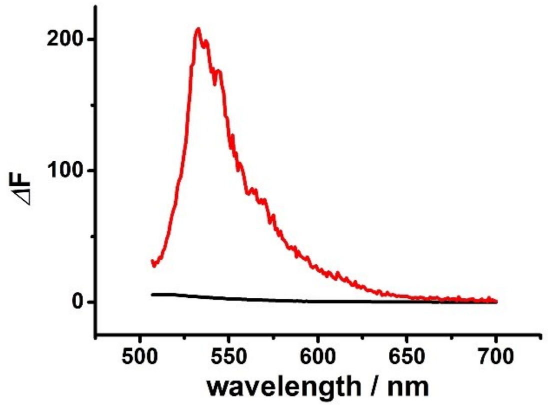

Figure S5. Fluorescence spectrum of the fluorescein-loaded NMOFs (red curve). The Fl-loaded NMOFs were precipitated and washed for several times. Then the fluorescence spectrum of the supernatant solution was recorded (black curve). It demonstrated that there is no leakage from the NMOFs since the poor fluorescence from the supernatant solution of NMOFs.

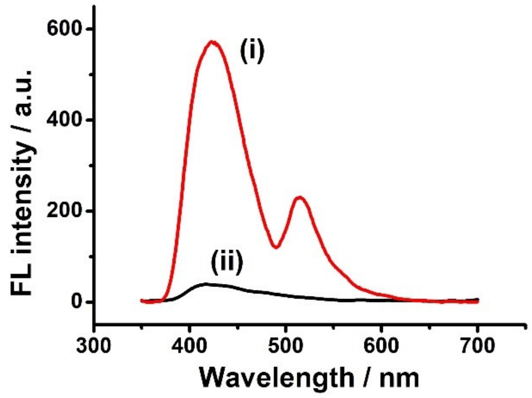

Figure S6. Fluorescence spectrum of the Fl-loaded NMOFs (red curve) upon sensing 250 nM of miRNA-155 (curve i) and 250 nM of miRNA-145 (curve ii). This result demonstrates that treatment of the NMOFs with foreign miRNA, miRNA-145, did not lead to any CRET signals.

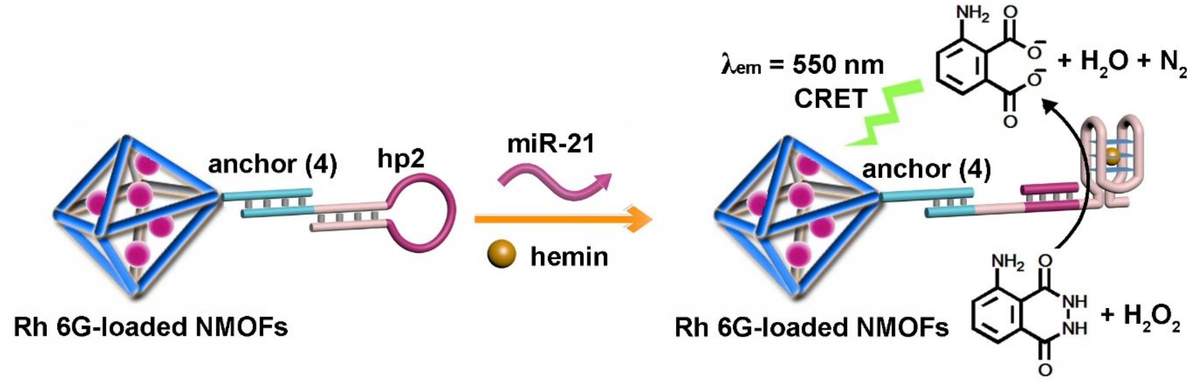

Figure S7. Rh 6G-loaded hairpin hp2-functionalized UiO-66 NMOFs for the CRET analysis of miRNA-21. The miRNA-21-triggered opening of the hairpin leads, in the presence of K^+-ions and hemin, to the self-assembly of the hemin/G-quadruplex that catalyzes the generation of chemiluminescence, $\lambda_{em} = 420$ nm, and the CRET-induced fluorescence of Rh 6G, $\lambda_{em} = 550$ nm.

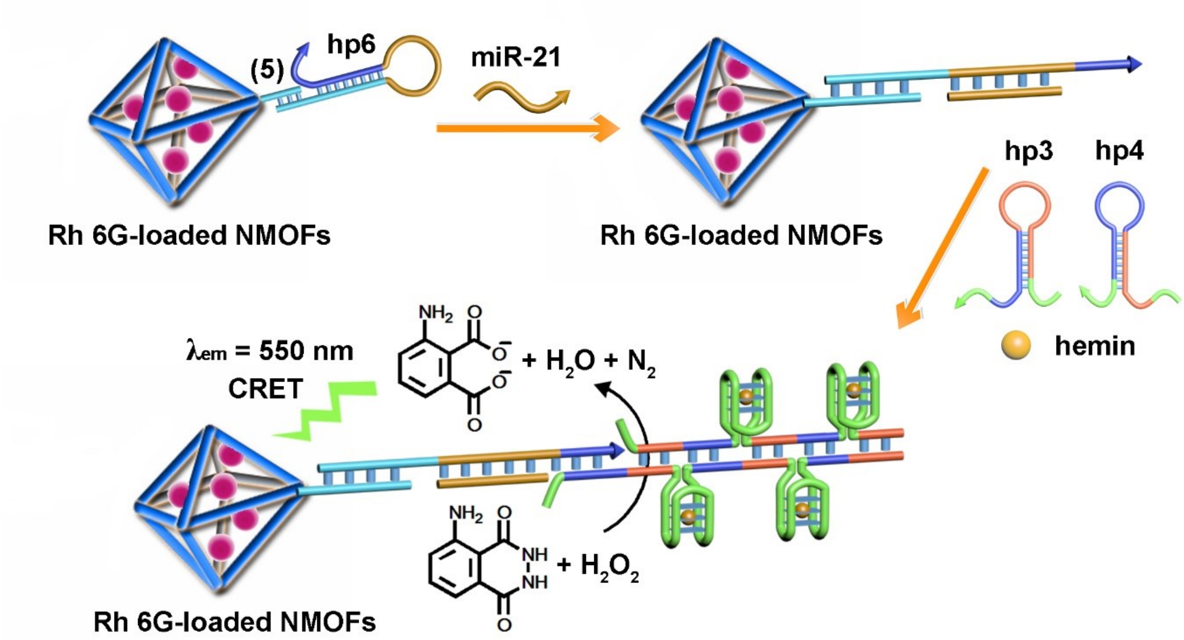

Figure S8. Amplified CRET-induced detection of miRNA-21 by coupling the HCR generating hemin/G-quadruplex polymers to the Rh 6G-loaded NMOFs.

Multiplexed analysis of the two miRNAs by hairpin-functionalized NMOFs.

The loading of the UiO-66 NMOFs with two different fluorophores and the gating of the NMOFs with two different hairpin gates allowed then the parallel multiplexed analysis of the two miRNAs, Figure S9. The mixture of the two NMOFs allows the CRET detection of the individual miRNA-155, Panel A, the miRNA-21, Panel B, and the CRET signals of the two dyes in the presence of the two miRNAs at $\lambda = 520$ nm and $\lambda = 550$ nm, respectively, Panel C. To effectively perform the multiplexed analysis of miRNA-155 and miRNA-21, and due to the difference in the CRET readout signals of the two fluorephores, we applied a 1:3 ratio of the Fl-loaded NMOFs and the Rh6G-loaded NMOFs.

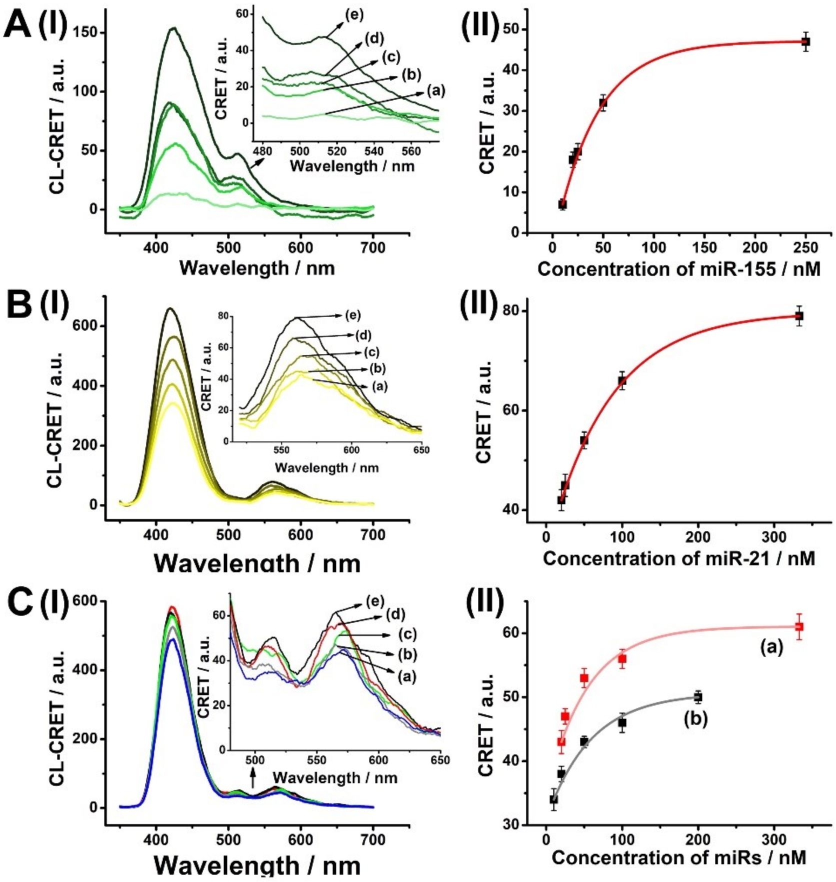

Figure S9. Multiplexed analysis of the miRNA-155 and miRNA-21 by a mixture consisting of the Fl-loaded hp1-modified NMOFs and the Rh 6G-loaded hp2-modified NMOFs using the hairpin-functionalized NMOFs CRET-induced analysis of the two miRNAs upon: Panel A-

Subjecting the mixture to variable concentrations of miRNA-155: (a) 10 nM; (b) 20 nM; (c) 25 nM; (d) 50 nM; (e) 250 nM. Panel B-Subjecting the mixture to variable concentration of BRCA1: (a) 20 nM; (b) 25 nM; (c) 50 nM; (d) 100 nM; (e) 333 nM. Panel C-Subjecting the mixture to variable concentrations of miRNA-155: (a) 10 nM; (b) 20 nM; (c) 50 nM; (d) 100 nM; (e) 200 nM, cuve (b) in panel II, and to variable concentrations of miRNA-21: (a) 20 nM; (b) 25 nM; (c) 50 nM; (d) 100 nM; (e) 333 nM, curve (a) in panel II. Error bars derived from N=3 experiments.

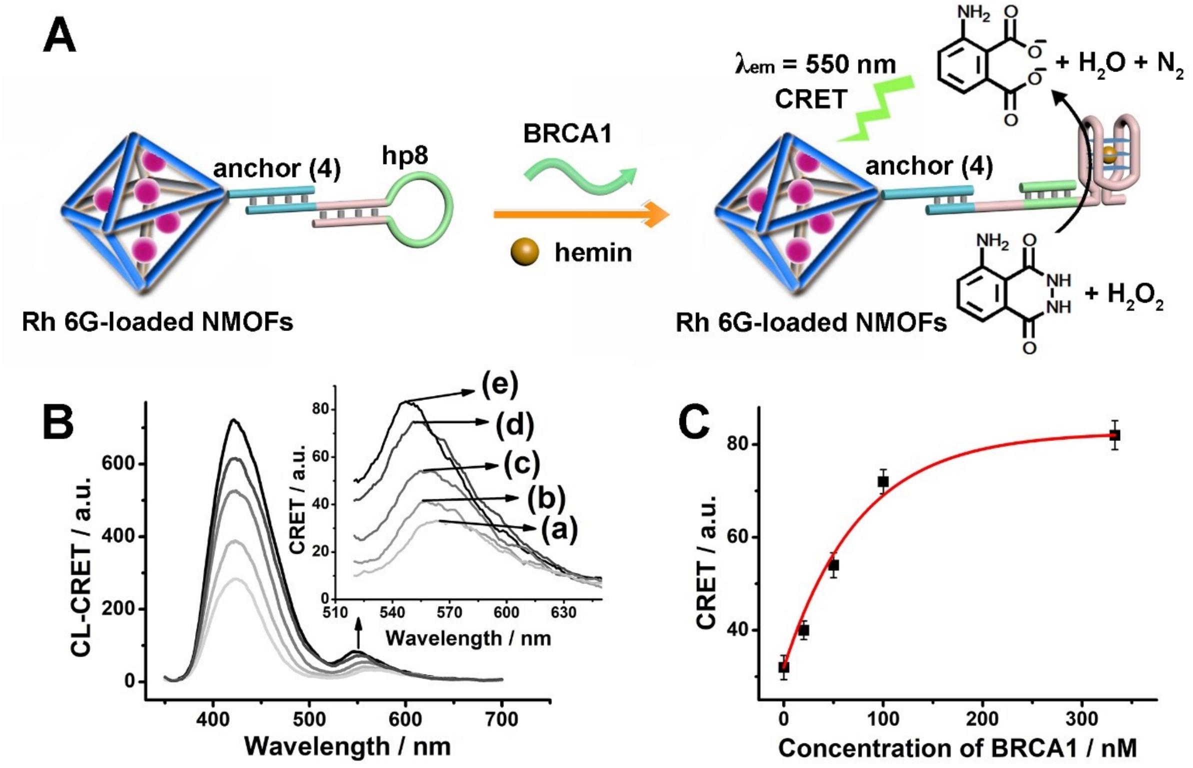

Figure S10. (A) Rh 6G-loaded hairpin hp8-functionalized UiO-66 NMOFs for the CRET analysis of gene BRCA1. The BRCA1-triggered opening of the hairpin leads, in the presence of K^+-ions and hemin, to the self-assembly of the hemin/G-quadruplex that catalyzes the generation of chemiluminescence, λ_{em} = 420 nm, and the CRET-induced fluorescence of Rh 6G, λ_{em} = 550 nm. (B) CRET-induced fluorescence spectra of Rh 6G, λ_{em} = 550 nm, in the presence of variable concentrations of BRCA1: (a) 333 nM; (b) 100 nM; (c) 50 nM; (d) 25 nM; (e) 20 nM. (C) Derived calibration curve corresponding to the CRET fluorescence signals of Rh 6G generated at variable concentrations of BRCA1. Error bars derived from *N*=3 experiments.

Multiplexed analysis of the two genes by hairpin-functionalized NMOFs.

A mixture of the hp7 and hp8 modified NMOFs allowed the multiplexed analysis of the genes, Figure S11. In the presence of p53, only the CRET signal of Fl at $\lambda = 520$ nm is observed, panel A, and in the presence of the BRCA1, the fluorescence of only Rh6G, $\lambda = 550$ nm, is observed, panel B. In the presence of the two genes, the CRET signals of Fl and Rh6G are detected, panel C. Note that, due to the different CRET signals of the fluorophores, the mixture of the Fl/Rh6G-loaded NMOFs was used with the ratio of 1:3 to obtain a clear separation of the CRET signals generated by the two fluorophores.

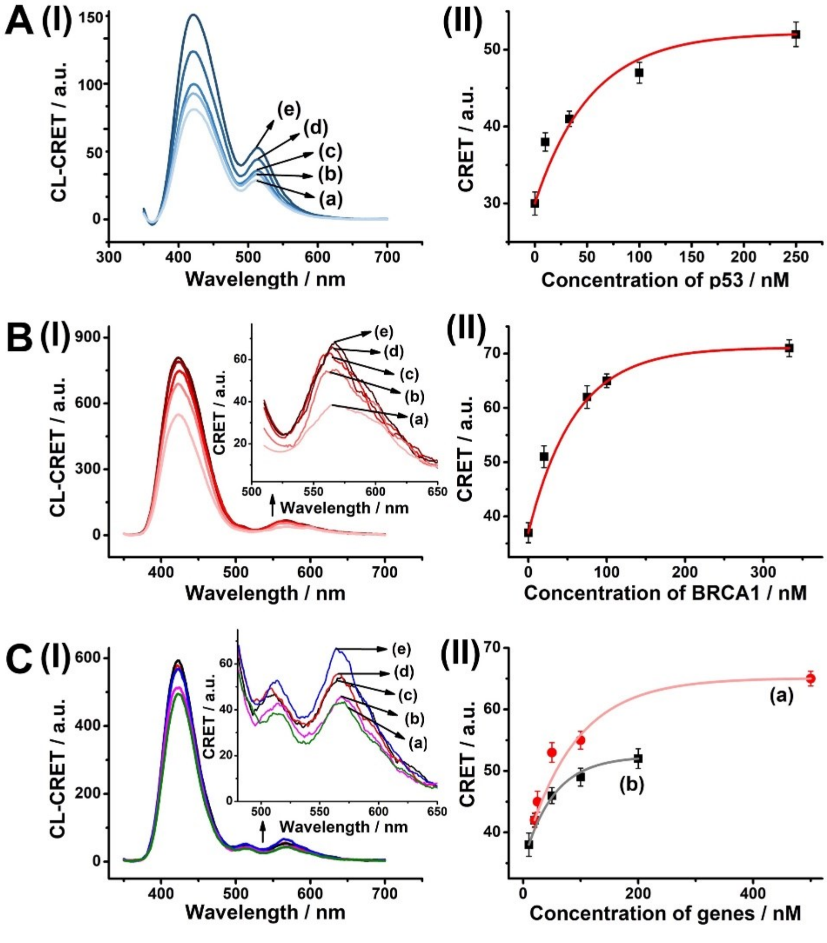

Figure S11. Multiplexed analysis of the p53 and BRCA1 by a mixture consisting of the Fl-loaded hp7-modified NMOFs and the Rh 6G-loaded hp8-modified NMOFs using the hairpin-functionalized NMOFs CRET-induced analysis of the two genes upon: Panel A-Subjecting the mixture to variable concentrations of p53: (a) 0 nM; (b) 10 nM; (c) 33 nM; (d) 100 nM; (e) 250

nM. Panel B-Subjecting the mixture to variable concentrations of BRCA1: (a) 0 nM; (b) 20 nM; (c) 75 nM; (d) 100 nM; (e) 333 nM. Panel C-Subjecting the mixture to variable concentrations of p53: (a) 0 nM; (b) 10 nM; (c) 50 nM; (d) 100 nM; (e) 200 nM, curve (b), and to variable concentrations of BRCA1: (a) 0 nM; (b) 20 nM; (c) 50 nM; (d) 100 nM; (e) 500 nM, curve (a). Error bars derived from N=3 experiments.
